# Multiphoton imaging to identify grana, stroma thylakoid, and starch inside an intact leaf

**DOI:** 10.1186/1471-2229-14-175

**Published:** 2014-06-27

**Authors:** Mei-Yu Chen, Guan-Yu Zhuo, Kuan-Chieh Chen, Pei-Chun Wu, Tsung-Yuan Hsieh, Tzu-Ming Liu, Shi-Wei Chu

**Affiliations:** 1 Department of Physics, National Taiwan University, Taipei, Taiwan; 2 Institute of Biomedical Engineering, National Taiwan University, Taipei, Taiwan; 3 Molecular Imaging Center, National Taiwan University, Taipei, Taiwan

**Keywords:** Grana, Starch, Two-photon fluorescence, Second harmonic generation

## Abstract

**Background:**

Grana and starch are major functional structures for photosynthesis and energy storage of plant, respectively. Both exhibit highly ordered molecular structures and appear as micrometer-sized granules inside chloroplasts. In order to distinguish grana and starch, we used multiphoton microscopy, with simultaneous acquisition of two-photon fluorescence (2PF) and second harmonic generation (SHG) signals. SHG is sensitive to crystallized structures while 2PF selectively reveals the distribution of chlorophyll.

**Result:**

Three distinct microstructures with different contrasts were observed, i.e. “SHG dominates”, “2PF dominates”, and “SHG collocated with 2PF”. It is known that starch and grana both emit SHG due to their highly crystallized structures, and no autofluorescence is emitted from starch, so the “SHG dominates” contrast should correspond to starch. The contrast of “SHG collocated with 2PF” is assigned to be grana, which exhibit crystallized structure with autofluorescent chlorophyll. The “2PF dominates” contrast should correspond to stroma thylakoid, which is a non-packed membrane structure with chrolophyll. The contrast assignment is further supported by fluorescence lifetime measurement.

**Conclusion:**

We have demonstrated a straightforward and noninvasive method to identify the distribution of grana and starch within an intact leaf. By merging the 2PF and SHG images, grana, starch and stroma thylakoid can be visually distinguished. This approach can be extended to the observation of 3D grana distribution and their dynamics in living plants.

## Background

**Figure 1:**
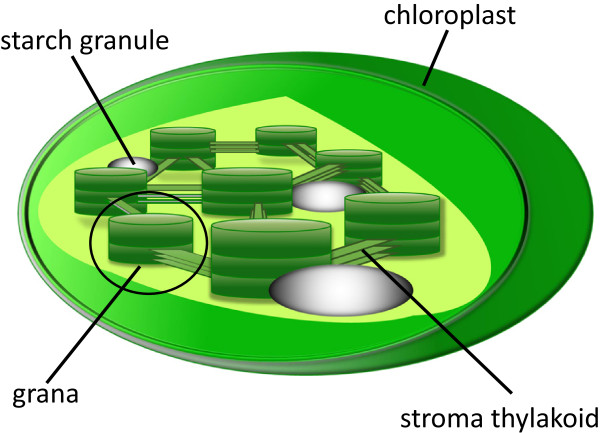
**Sub-organelles inside a chloroplast.** Grana and stroma thylakoid contains fluorescent chlorophyll. Both starch granules and grana exhibit stacking structures, allowing SHG conversion.

Photosynthesis is one of the most important chemical reactions in the world. Based on the photochemical effect of chlorophyll, it can convert light into chemical energy and store it in the bonds of sugar. During photosynthesis of plants, light reaction occurs in thylakoid, which contains chlorophyll *a*, chlorophyll *b*, and carotenoid to carry out photochemical interactions. Inside a chloroplast, thylakoid has two structural forms. One is stroma thylakoid (stroma lamellae), which is a membrane-bound structure embedded into the stroma of chloroplast. The other is granum, which is a well-packed stack of thylakoids (see Figure 1). Grana are the major functional structures for photosynthesis, and are interconnected by stroma thylakoid, which does not exhibit crystallized structure. The product of photosynthesis is subsequently transformed into polysaccharides for storage. The main polysaccharide form of storage is starch, which comprises stacks of crystallized amylopectin and amorphous amylose.

The three-dimensional arrangement of grana and starch granules in a chloroplast has been observed by transmission electron microscopy (TEM) and scanning electron microscopy (SEM) [1-6]. As expected, TEM and SEM provide very high spatial resolution down to nanometer scale. Nevertheless, both TEM and SEM need sophisticated specimen preparation. TEM specimens cannot be thicker than hundreds of nanometers, so it is not practical for TEM to perform whole-leaf observation. For SEM, since the chamber is at high vacuum, plant specimen is normally required to be fully dehydrated by chemical fixation, which may cause molecular denaturation and structure artifact. The freeze-fracture approach for SEM sample processing might authentically display the fine structures of the chloroplast, but it cannot be used in live cell applications.

Optical microscopy, on the other hand, exhibits much lower spatial resolution (about half of visible wavelength), but it can be used to view cells that are living and functioning. Conventional compound microscopes are not capable of producing a clear three-dimensional view in thick biological tissues. By combining a confocal pinhole with laser scanning microscopy, confocal microscopy provides excellent optical sectioning capability. In the late 1980s, confocal microscopy was adopted to study the inner structures of chloroplasts in living and intact plants based on the autofluorescence from chlorophyll [7,8]. However, since both grana and stroma thylakoid contain chlorophyll, it is difficult to distinguish them simply by fluorescence intensity, unless using sophisticated spectral imaging microscope [9,10]. Since grana are well-packed thylakoids, a contrast agent that is sensitive to crystallization will be useful to distinguish grana and stroma thylakoids. One possible solution is second harmonic generation, which is a nonlinear optical process that is allowed only in non-centrosymmetric structures [11].

**Figure 2:**
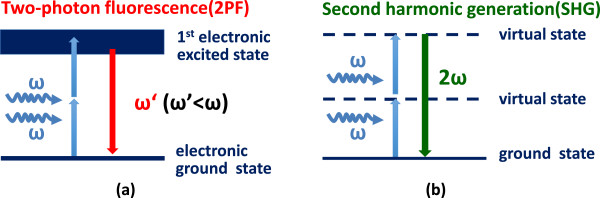
**Transition diagrams of 2PF and SHG. (a) **Two-photon excitation occurs through the absorption of two lower-energy photons via short-lived intermediate states. After excitation, the fluorophore first relaxes to the lowest energy level of the excited electronic states via fast vibrational processes, and then relaxes back to the ground state, emitting a spontaneous fluorescence photon. **(b)** In the process of SHG, two photons are annihilated and a single photon with doubled energy (half the wavelength) is generated. No real-state transition is required in SHG.

Nonlinear optical microscopy has become an important tool for imaging biological samples [12-14]. There are several different contrast mechanisms in nonlinear optical microscopy family, including two -photon fluorescence (2PF), second harmonic generation (SHG), coherent anti-Stokes Raman scattering (CARS), etc. Because of the nonlinear dependence between signal and excitation, one of the main advantages of nonlinear optical microscopy lies in its intrinsic optical sectioning capability. Since there is no pinhole required, the detection efficiency is greatly enhanced. In addition, infrared laser is typically used as the excitation source, significantly increasing the penetration depth due to the reduced scattering in biological tissues. In our study, we focus on 2PF and SHG, which are sensitive to autofluorescence of chlorophyll and crystallization, respectively, to identify grana.Two-photon excitation is a process in which a fluorophore is excited by simultaneous absorption of two photons. This nonlinear process can occur if the sum of the energies of the two photons matches the energy gap between the ground and excited states of a molecule. 2PF differs from traditional fluorescence microscopy in which the excitation wavelength is longer than the emission wavelength, and the summed energies of two long-wavelength exciting photons will produce a shorter emission wavelength, as shown in Figure 2(a). The advantages of 2PF microscopy over conventional single photon fluorescence are mainly the increased penetration depth and intrinsic optical sectioning. But both 2PF and single photon fluorescence requires real state transition, which means part of the excitation energy is inevitably deposited inside the sample.

In the case of SHG, laser light is focused on a sample to generate frequency-doubled light. During SHG process, two incident photons are annihilated and a new photon is generated. Due to energy conservation, the energy, and thus frequency, of the new photon is twice of that of the annihilated photons, as shown in Figure 2(b). One advantage of SHG is that only virtual state transition is involved, thus no photo-damage and photon-bleaching are expected, since no energy is deposited during this transition [15]. On the other hand, SHG also exhibits a square dependence on incident intensity, so SHG imaging provides excellent optical sectioning capability, similar to 2PF imaging. In principle, SHG is sensitive to non-centrosymmetric crystallized media, such as an interface between two centrosymmetric media [16], and crystallized structures, such as well-packed biological structures, including collagen and myosin in animal, as well as starch and grana in plant [11,16-27]. Inside a chloroplast, both starch and grana exhibit SHG, so in a previous work [28], the authors identified SHG of grana by keeping the plants in the dark for approximately 3 weeks to devoid starch content in the plant. However, this process might severely affect plant physiology, and the method prevents study of photosynthesis under normal illumination condition.

The aim of this paper is to demonstrate that nonlinear optical microscopy is a noninvasive method to identify the distribution of grana, stroma thylakoid, and starch granules within a live intact leaf. No complicated sample preparation or darkroom process is required. Through simultaneous acquisition of 2PF and SHG signals, these cellular organelles can be visually identified. This novel approach can be used in the field of botanical evolutionism, and provides a dynamic imaging observation in the growth of the plants.

## Results

**Figure 3:**
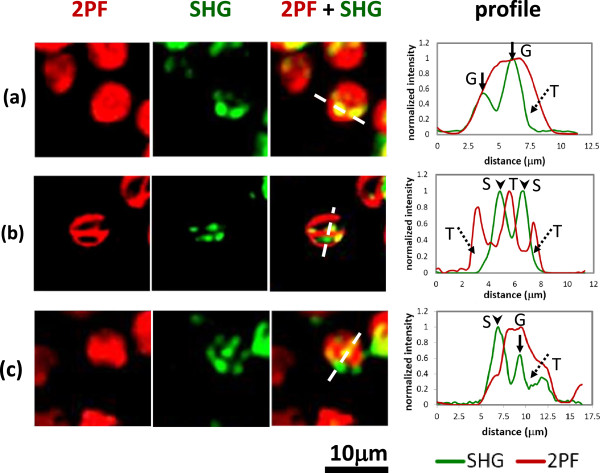
**Multiphoton laser scanning microscopic images of chloroplasts inside an intact leaf.** The backward 2PF and forward SHG signals are presented as red and green colors, respectively. We choose three different regions to illustrate the distribution and meaning of colors. For each region, a line profile corresponds to the white dashed line is shown to the right. (arrow: grana; arrowhead: starch; dotted arrow: stroma thylakoid) **(a)** The chloroplast is filled with 2PF (red) overlapped with discrete SHG (green) spots. In the combined image, the red part corresponds to stroma thylakoid, and the yellow (=green + red) part corresponds to grana. **(b)** The distribution of 2PF is complementary to SHG. In the combined image, the red part still corresponds to stroma thylakoid, and the green part shows the location of starch granules. **(c)** In the combined image, yellow, green, and red correspond to grana, starch, and stroma thylakoid, respectively.

Figure 3 shows distribution of 2PF (red) and SHG (green) images in the chloroplasts that are within live leaves. The backward 2PF and forward SHG signals are simultaneously recorded using the multiphoton scanning microscope (see Methods). Roughly speaking, individual chloroplast can be outlined by the 2PF signal, but obviously, both 2PF and SHG are not evenly distributed inside a chloroplast. In Figure 3(a), 2PF fills most part of the chloroplast while SHG shows discrete spots. By overlapping the two images and plotting a line profile, it is clear that these SHG spots are collocated with 2PF, showing the color of yellow in the image. So the yellow parts indicate the co-existence of chlorophyll autofluorescence and stacked structure, corresponding to grana. For those regions with 2PF but no SHG, they exhibit red color and should correspond to stroma thylakoid, which exhibit autofluorescence but no stacked structure. The detailed intensity distribution of SHG and 2PF along the dashed line is given in the right panel of Figure 3(a).

In Figure 3(b), the distribution of SHG and 2PF are complementary to each other. As can be seen in the 2PF image, there are cavities within each chloroplast. In the SHG image, there are multiple bright spots showing the location of crystallized structures. In the combined image, as well as in the line profile, we see that the 2PF cavities are filled with bright SHG spots. Again, for those regions in red color, they represent stroma thylakoid. On the other hand, for those regions with SHG but no 2PF, they exhibit green color and should correspond to crystallized structures without autofluorescence in chloroplast, i.e. starch [11,23].In Figure 3(c), some SHG spots are collocated with 2PF, while others are not. Based on previous discussion, the green spots (SHG dominates) indicate the location of starch granules; the yellow color (SHG collocated with 2PF) provides the distribution of grana, while the red color (2PF dominates) corresponds to stroma thylakoid. It is interesting to notice that typically chloroplasts are in a round to elliptical shape, but the 2PF image here shows an irregular outline. It has been known from the electron microscopy studies that stroma thylakoid is not evenly distributed inside chloroplast, so the fact that 2PF is weak in some area reflects the low concentration of stroma thylakoid.

In addition, an axial image series (see Additional file 1) is given in the movie, demonstrating excellent optical sectioning capability of nonlinear optical microscopy in plant leaves. Based on the discussion above, the distribution of grana, starch, and stroma thylakoid can be identified by color of yellow, green, and red, respectively.

## Discussion

Chloroplast is the main organelle for photosynthesis. Inside a chloroplast, grana and stroma thylakoids are the key biological structures to convert light energy into chemical energy, which is stored in the form of starch granules. Here we demonstrated to visualize the distribution of grana, stroma thylakoids, and starch granules, in individual chloroplast within an intact leaf based on multiphoton laser scanning microscopy, which provides three-dimensional sub-micrometer resolution. From our study, since both grana and stroma thylakoid contains chlorophyll, they exhibit strong 2PF signals. On the other hand, the SHG contrast of multiphoton imaging is sensitive to stacked (crystallized) structure, so SHG image reveals the distribution of grana and starch granules, which are the only two organelles inside a chloroplast that emit SHG. By combining both 2PF and SHG modalities, the signal from grana, starch, and stroma thylakoid, can be well separated visually, as summarized in Table 1.

**Table 1:** The table gives a summary of 2PF and SHG signals correspond to three different kinds of structures, including grana, starch and stroma thylakoid

**Signal/structure**	**Grana**	**Starch**	**Stroma thylakoid**
2PF	Yes	No	Yes
SHG	Yes	Yes	No
Color	Yellow	Green	Red

One possible ambiguity lies in the interpretation of overlapped SHG and 2PF. Our current explanation is that the overlapping reflects the coexistence of chlorophyll autofluorescence and stacked structures, i.e. grana. However, it is possible that the structure we assigned to be a granum might be a starch granule surrounded by stroma thylakoid. Here we describe the reason why the latter interpretation is more unlikely.Let's examine Figure 3(a) more closely. Based on our experimental condition (objective and wavelength), the lateral and axial resolutions are better than 0.5 and 1 μm, respectively. The size of a granum and a starch granule in our sample should be about 1 μm, since we used a shade plant (see Methods). The sizes of SHG spots are larger than 1 μm in Figure 3(a). If these SHG spots are large starch granules (i.e. larger than 1 μm) surrounded by stroma thylakoid, the 2PF signals should significantly drop at the center of the SHG spots. Nevertheless, we did not observe this in Figure 3(a). On the other hand, it might be a very tiny starch granule that generate SHG but overlapped with 2PF from surrounding stroma thylakoid. However, it is also unlikely because the SHG spots we observed in Figure 3(a) are more than 1 μm in width, which is larger than the resolution limit of our multiphoton system. It is evident that we can distinguish the orientation of the two selected SHG microparticles in Figure 3(a), and it serves as a proof that the optical resolution is better than the size of the microparticles. In fact, from the intensity profiles of Figure 3(a), the fluorescence signals actually increase along with SHG in the particles. So we conclude that the selected SHG particles in Figure 3(a) should correspond to grana, not starch granules.On the other hand, for the structure that we assigned to be a starch granule, could it be a granum? The first thing to note is that fluorescence intensity reflects local density of chlorophyll, and the volume density of chlorophyll is higher in grana compared to surrounding stroma thylakoid. So the fluorescence intensity of grana should be no less than that of stroma thylakoid. As shown in Figure 3(b), if the SHG spots correspond to grana, the 2PF intensity at the location of SHG spots should be stronger or at least equal to the surrounding 2PF intensity. However, what we found in Figure 3(b) are particles with strong SHG and reduced 2PF signals. So they should be starch granules, not grana. The residual weak 2PF signals at the SHG spot might come from the stroma thylakoid adjacent to the starch granule in the axial direction.From Figure 3(c), in the region of grana, both 2PF and SHG increase compared to the surrounding; while in the region of starch, a strong SHG peak is observed with significantly reduced 2PF. This gives strong support that our technique can indeed separate starch and grana.

**Figure 4:**
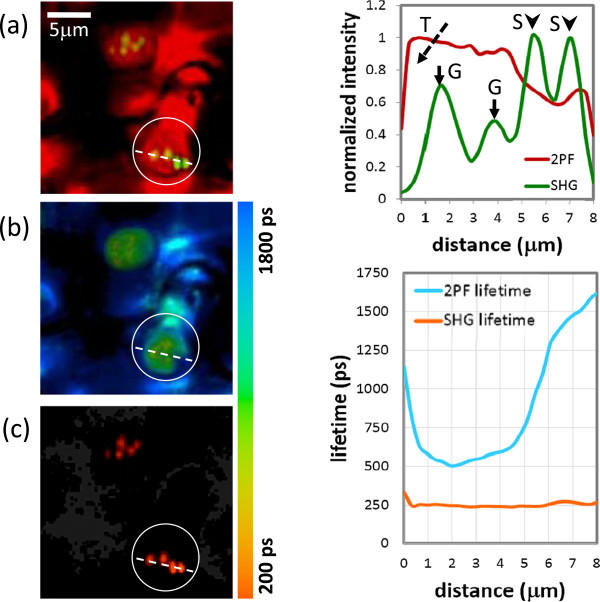
**Multiphoton imaging and fluorescence lifetime imaging of 2PF and SHG.** The intensity profile and lifetime profile corresponding to the dashed line in the highlighted area are shown in the right-hand side. **(a)** Merged images of SHG and 2PF signals, similar to those in Figure 3 **(b)** and **(c)***In-situ* measured 2PF and SHG lifetime imaging, respectively. The color bar shows the corresponding lifetime.

In order to confirm the discrimination among stroma thylakoid, grana, and starch granules, we have performed additional fluorescence lifetime imaging measurement. It is known that fluorescence of chloroplast comes mainly from photosystems (PS), including PSI and PSII. About 85% of PSII is located in grana, while PSI is dominating in stroma thylakoid [29,30]. Nevertheless, since the fluorescence spectra of PSI and PSII are largely overlapped to each other, it is not easy to distinguish them by simple spectral filtering. In terms of fluorescence lifetime, the dominating components of PSI and PSII are about 500 ps [31,32] and 1400 ps [29,30], respectively, so it is possible to distinguish grana and stroma thylakoid with lifetime measurement. On the other hand, the lifetime of SHG is extremely short due to its virtual transition nature, so it is also feasible to identify crystallized structure, i.e. grana and starch, via lifetime measurement.The lifetime measurement is shown in Figure 4. Figure 4(a) shows a merged image of 2PF and SHG signal intensities. As described in the preceding paragraphs, stroma thylakoid, starch, and grana correspond to red, green, and yellow colors, respectively. In the figure, most region is red, while in the highlighted area (a white circle), two grana (middle of the circle) and two starch granules (bottom right of the circle) can be found. A line profile corresponds to the dashed line is provided in the right side of Figure 4(a), showing again the variation of 2PF/SHG signals and their correspondence to different structures.Figure 4(b) and (c) presents the lifetime measurement of 2PF and SHG, respectively, in the same region. Similarly, a line profile showing lifetime distributions of 2PF and SHG along the dashed line are given in the right side of Figure 4(b) and (c). At first glance, it might be surprising that the image and the lifetime profile are mismatched. For example, four SHG emitters along the broken line in Figure 4(c), including grana and starch granules, can be clearly identified in the imaging mode, but not in the lifetime profile (orange line). This is because in the lifetime images, color presents the lifetime, and the brightness corresponds to signal intensity. However, in the right-hand-side the lifetime profile, only the information of lifetime is shown.In Figure 4(b), it is obvious that in most regions, the fluorescence lifetime is longer than 1200 ps, while in the highlighted area, the fluorescence lifetime is significantly shorter than 1000 ps. From the detailed comparison of line profiles of intensity and lifetime in Figure 4, apparently, in the region of grana (marked as G in the intensity profile), the corresponding 2PF lifetime (blue line) is close to 500 ps, manifesting the higher concentration of PSII. The 2PF lifetime increases dramatically in the region outside grana, showing that PSI is dominating, reflecting the existence of stroma thylakoid.For lifetime of SHG signals, as shown by Figure 4(c) and the orange line in the lifetime profile, it is indeed very short as expected, and is limited by the ~ 240 ps instrument response function (IRF) of our fluorescence lifetime imaging system. In the region of starch granules (marked as S in the intensity profile), strong SHG and weak 2PF are observed. With the support of lifetime information, now we know the 2PF in this region corresponds to stroma thylakoid. Therefore, more confidence is endorsed to the assignment of starch granule.In the upper part of Figure 4(a), there is another chloroplast with several grana inside. Similarly, in the corresponding region of Figure 4(b), the fluorescence lifetime becomes smaller due to the higher concentration of PSII. In the corresponding region of Figure 4(c), SHG spots with very short lifetime are observed, showing the existence of grana. In summary, the fluorescence lifetime measurements provide strong supporting evidence that our multiphoton approach (2PF + SHG) can indeed distinguish stroma thylakoid, grana, and starch granules.

## Conclusion

We have demonstrated a noninvasive method to identify the distribution of grana and starch inside a live mesophyll cell without any special specimen labeling or handling. The method is based on the combination of SHG and 2PF contrast in a multiphoton microscope. There are two types of SHG structures inside a chloroplast of a plant cell. One is collocated with strong 2PF, and the other is complementary to 2PF. The former correspond to grana while the latter correspond to starch. For those regions with only 2PF but no SHG, they represent the distribution of stroma thylakoid. By merging 2PF and SHG images, the grana, starch granules, and stroma thylakoid can be visually distinguished by different colors of yellow (green + red), green, and red, respectively. The structure identification is further proved by fluorescence lifetime measurements. The nonlinear nature of the multiphoton process provides useful intrinsic optical sectioning capability and is less likely to cause damage in live sample, enabling observation of organelle dynamics during plant growth. Our technique will be useful to study granal structural variation among different plant specie [33], and can be used in the field of botanical evolutionism.

## Methods

The leaf we used here was detached from a fresh ferns, *Macrothelypteris torresiana (Gaud.) Ching*, which belongs to shaded plants with large grana [33-37]. The leaf was mounted in water between a coverslip and a glass slide. The edges of the coverslip were sealed by nail varnish. The glass slide was placed on the microscope stage for observation.

**Figure 5:**
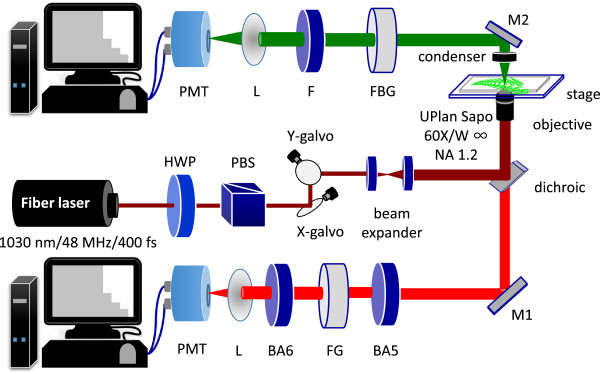
**Schematic diagram of the experiment set-up.** For intensity measurement, both backward and forward channels are used to detect 2PF and SHG signals, respectively. For lifetime measurement, a photon-counting PMT is placed in the forward direction, and connected to a TCSPC system. HWP: half-wave plate, PBS: polarization beam splitter, L: lens, BA6: BA610IF, FG: FGS600, BA5: BA565IF, F: FF520, FBG: FBG39. M1, M2: mirrors.

The experimental setup is shown in Figure 5, which is similar to our previous reports [23,38]. This setup allows the simultaneous measurement of SHG and 2PF in the forward and backward directions. The laser source is a mode-locked Yb:fiber laser, whose central wavelength is 1030 nm. The pulse width, repetition rate, and maximal average power are 400 fs, 48 MHz, and 5 W, respectively. The excitation light was directed into an Olympus FV300 system with a pair of X-Y galvanometric mirrors to achieve raster scanning. The pixel dwell time is 9 × 10^-6^ sec, and the acquisition time for one image (256 × 256 pixels) is about 0.6 sec. The excitation light was focused onto the specimen through the microscope objective lens (UPlanSApo 60×W, NA = 1.20, Olympus, Japan). The average laser power at sample position is about 60 – 70 mW. The 2PF signals were epi-collected by the same objective while the SHG signals were collected by a condenser in the forward direction. Two identical photomultiplier tubes (PMTs, R9110, Hamamatsu, Japan) with coolers were respectively placed in the forward and backward path to detect SHG and 2PF signals. There is a dichroic mirror (DM-BG, Olympus, Japan) inside FV300 to reflect IR and to allow the transmission of the 2PF signals. Additional color filters (FBG39 and FGS600, Thorlabs, NJ, USA) were placed in front of each PMT to ensure that laser is appropriately blocked. Filters for SHG (FF01-520/15-25, Semrock, NY) and for 2PF (BA565IF and BA610IF, Olympus, Japan) are inserted before corresponding PMTs to ensure only SHG and 2PF signals were recorded.

For fluorescence lifetime measurement, the excitation and scanning systems are the same as above, but the detection part becomes a photon-counting PMT (PMC-100-1, Becker & Hickl, Germany) in the forward direction, equipped with a time-correlated single photon counting system (TCSPC-150, Becker and Hickl, Germany). A high-speed photodetector synchronize the laser repetition rate to the photon counting system. During lifetime measurement, corresponding filters are placed in front of the photon counting PMT to allow either 2PF or SHG detection without cross talk.

## Authors’ contributions

MYC, GYZ, KCC, PCW, and TYH performed the experiments. MYC analyzed the data and wrote the manuscript with SWC. TML and SWC supervised the project and instruments. All authors read and approved the final manuscript.

## Supplementary Material

Additional file 1 **Z-stack merge image of *Macrothelypteris torresiana (Gaud.) Ching* leaf.** The chloroplasts are filled with 2PF (red) overlapped with discrete SHG (green) spots. Click here for file
